# Supplementary material for: Precursor B-ALL Cell Lines Differentially Respond to SYK Inhibition by Entospletinib
Source: Int J Mol Sci. 2021 Jan 8;22(2):592. doi: 10.3390/ijms22020592 (PMC7827334; doi:10.3390/ijms22020592)
Supplement: Supplementary file 1 [file ijms-22-00592-s001.zip › supplementary material/Supplementary file B - figures.docx]

# Supplementary file B

# Figures


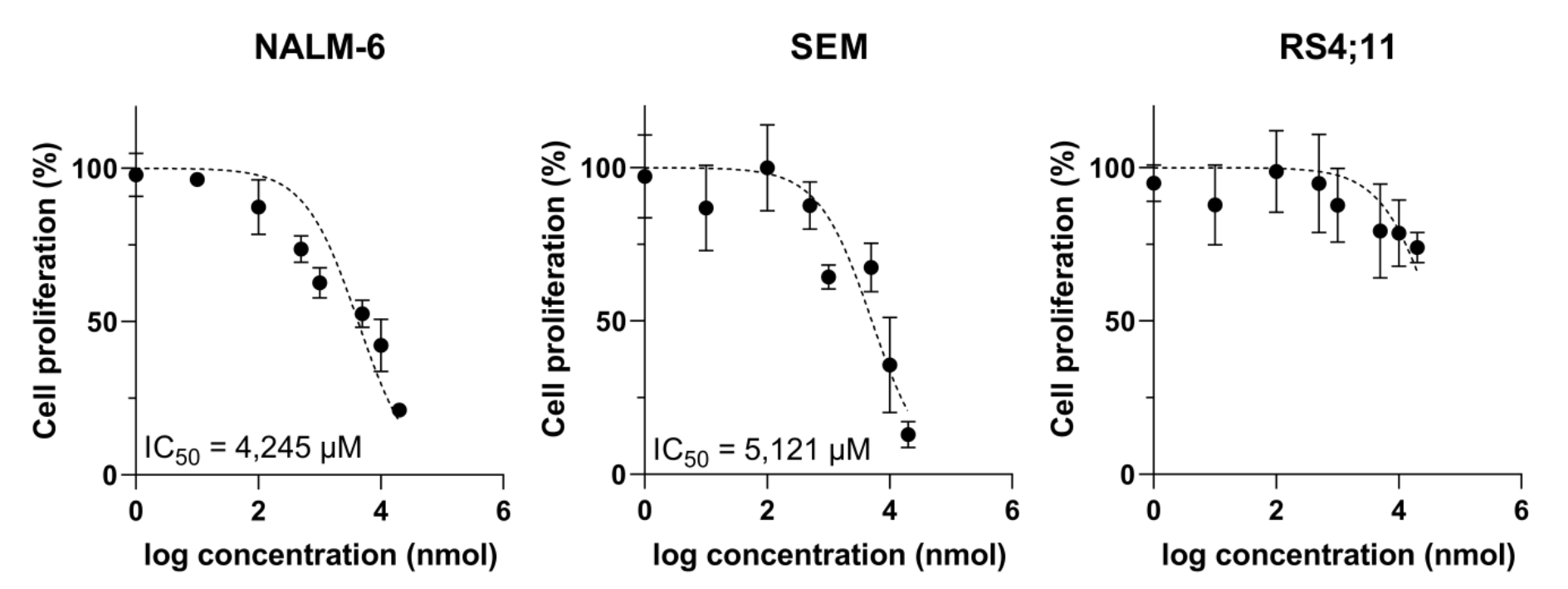


Figure S1: Entospletinib IC50 calculation

Concentration dependent reduction of cell proliferation in pre-B-ALL cell line NALM-6 and pro-B-ALL cell lines SEM and RS4;11 by Entospletinib. IC50 calculation based on cell proliferation, values are estimated by Graphpad prism software. IC50 for RS4;11 was not reached. n ≥ 3.


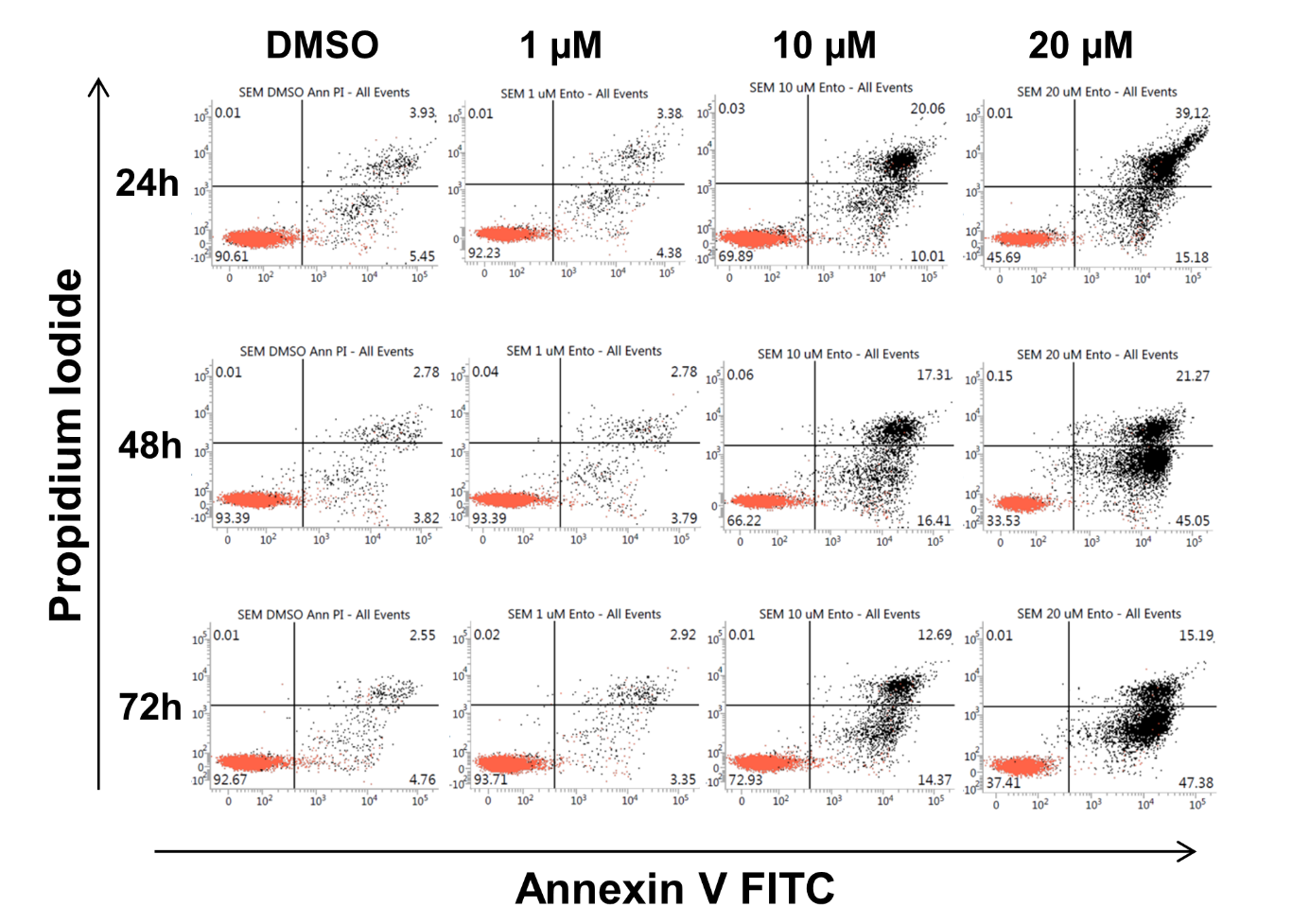


Figure S2: Apoptosis induction in B-ALL after Ento exposition

Concentration dependent apoptosis induction by Entospletinib in pro-B-ALL cell line SEM. Exemplarily apoptosis measurement of Ento exposed SEM cells at 24 h, 48 h and 72 h by flow cytometry.


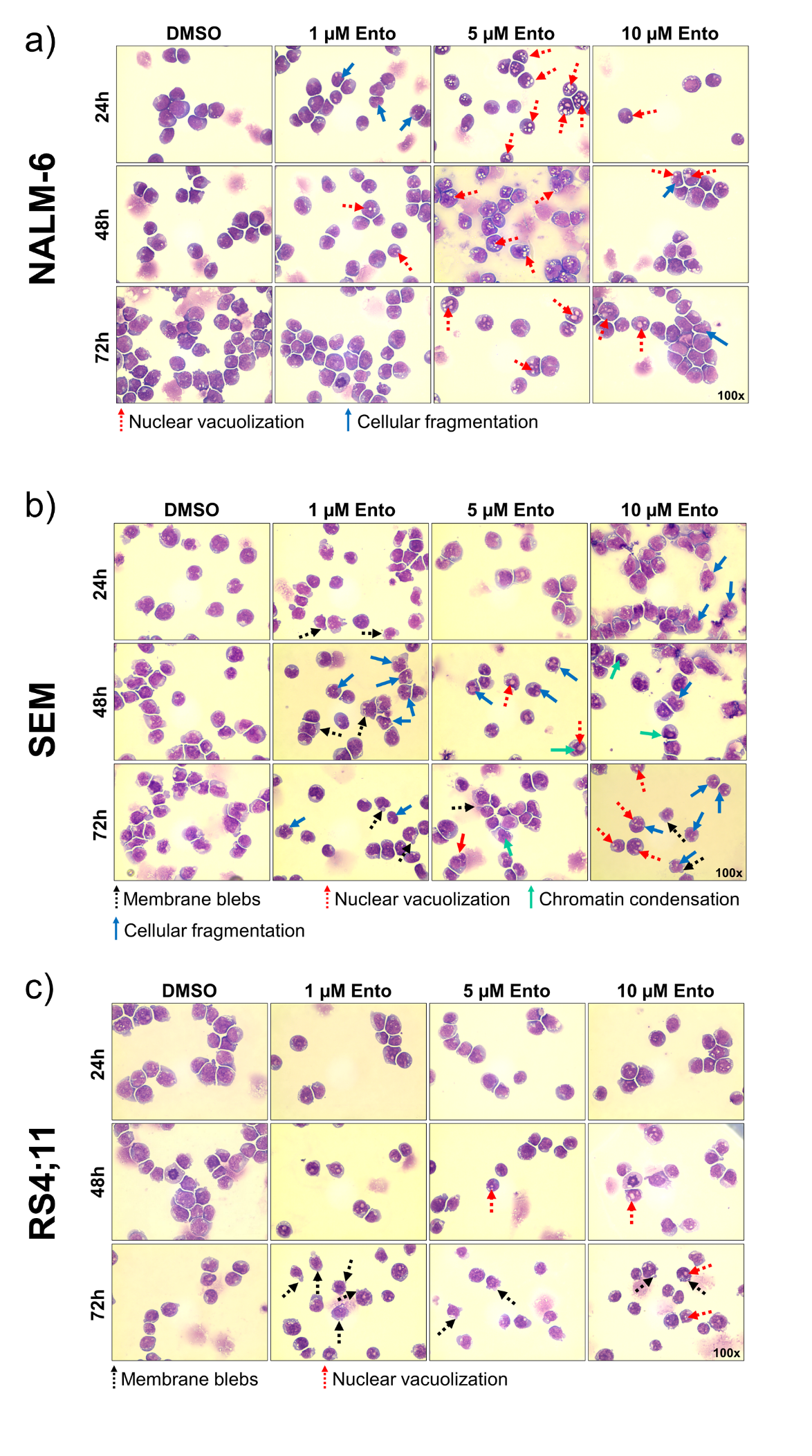


Figure S3: Morphological characterization after Entospletinib exposure

Light microscopy images (x 100) of Entospletinib exposed B-ALL cells revealed apoptosis induction in pre-B-ALL cell line NALM-6 and pro-B-ALL cell line SEM. Entospletinib induced only moderate morphological changes in pro-B-ALL cell line RS4;11. Cytospins were stained with May-Gruenwald Giemsa stain (Pappenheim method) after Entospletinib exposure at different concentrations (1 µM, 5 µM, 10 µM) and different time points (24 h, 48 h, 72 h).


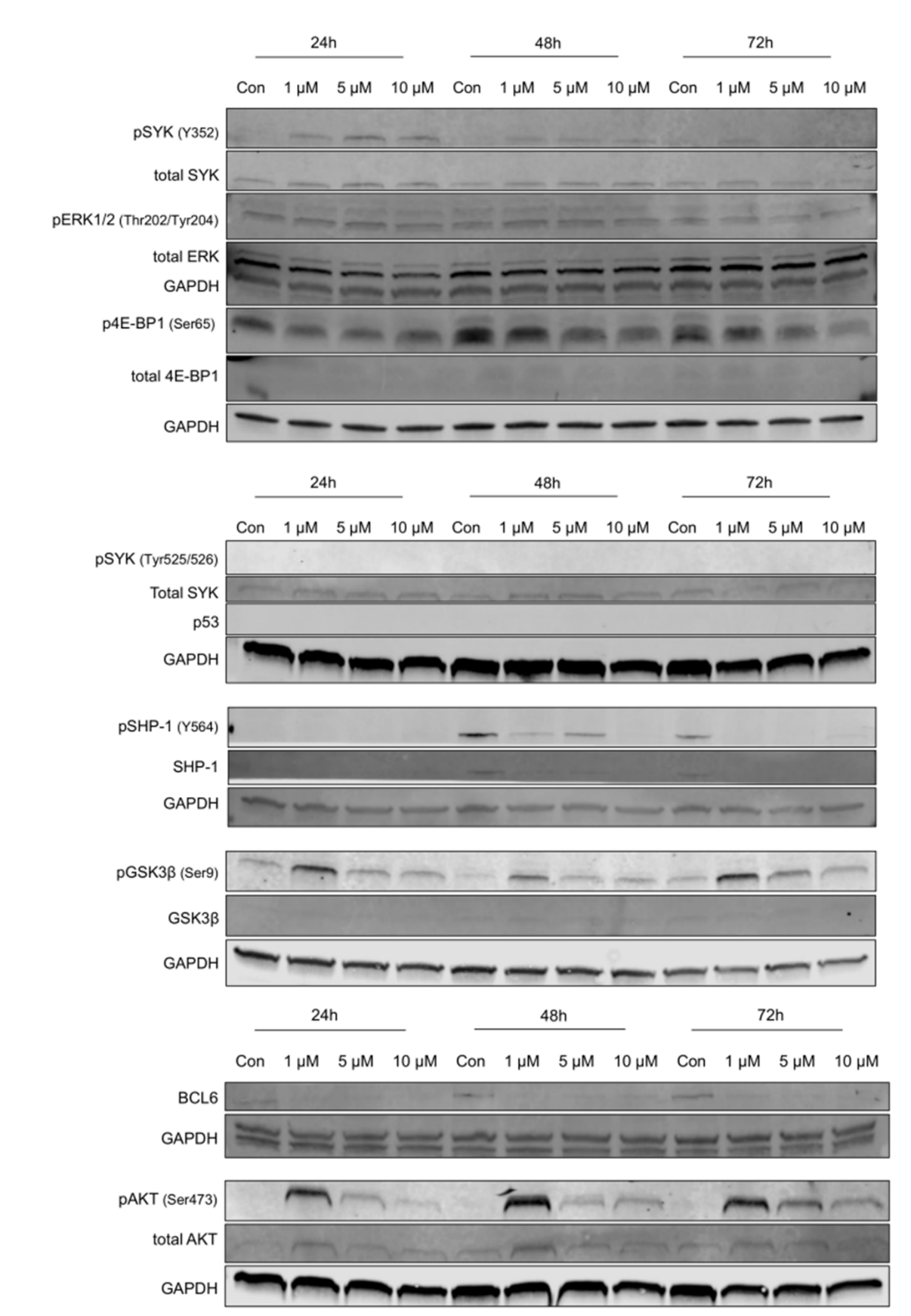


Figure S4: Entospletinib induced distinct downstream protein modifications in pre-B-ALL NALM-6

Western Blot analyses revealed distinct changes of SYK downstream proteins of in pre-B-ALL NALM-6. Representative Western Blot images of NALM-6 cells after 24 h, 48 h and 72 h Entospletinib exposure (n ≥ 3).


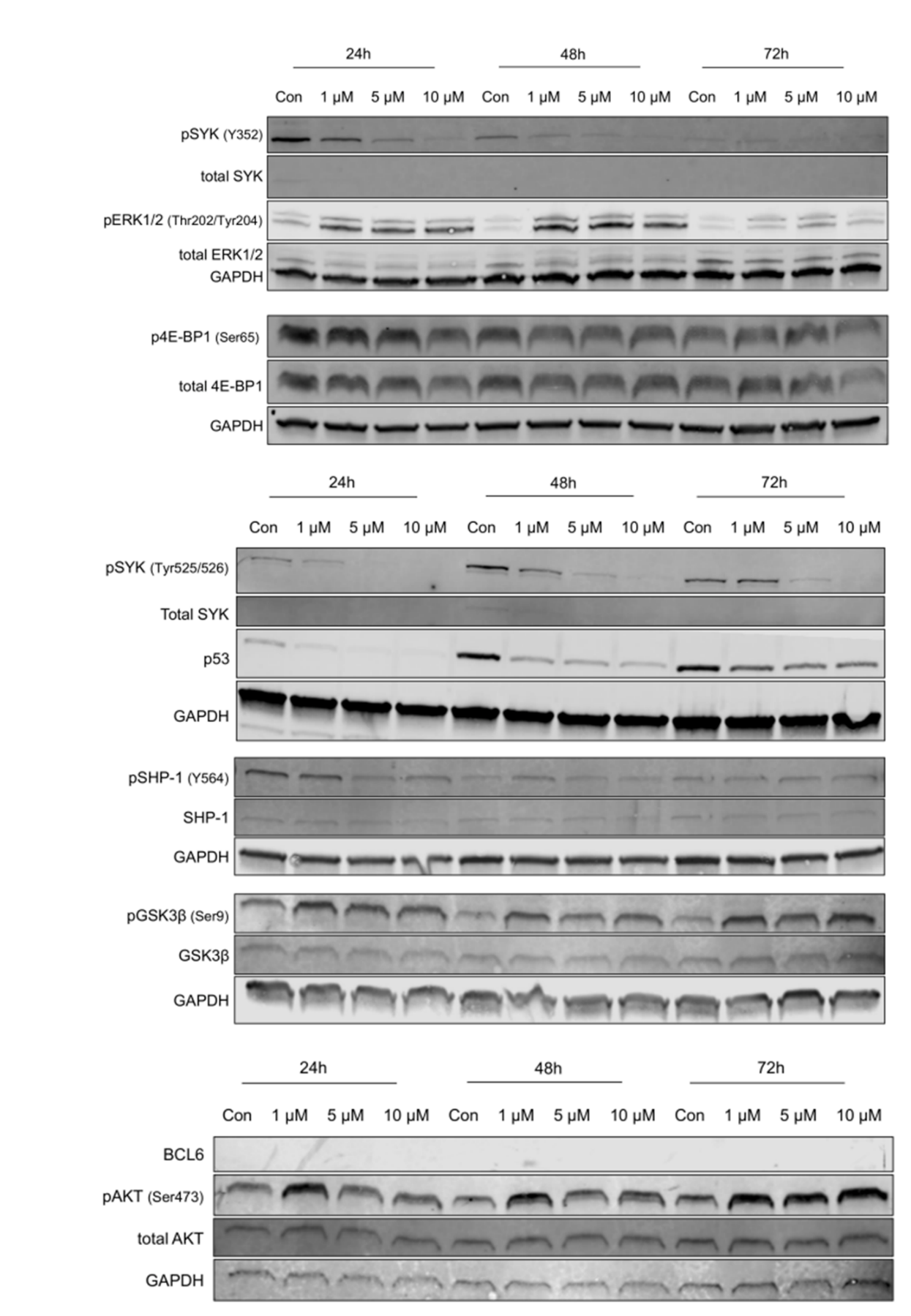


Figure S5: Entospletinib induced distinct downstream protein modifications in pre-B-ALL SEM

Western Blot analyses revealed distinct changes of SYK downstream proteins of in pro-B-ALL SEM. Representative Western Blot images of SEM cells after 24 h, 48 h and 72 h Entospletinib exposure (n ≥ 3).


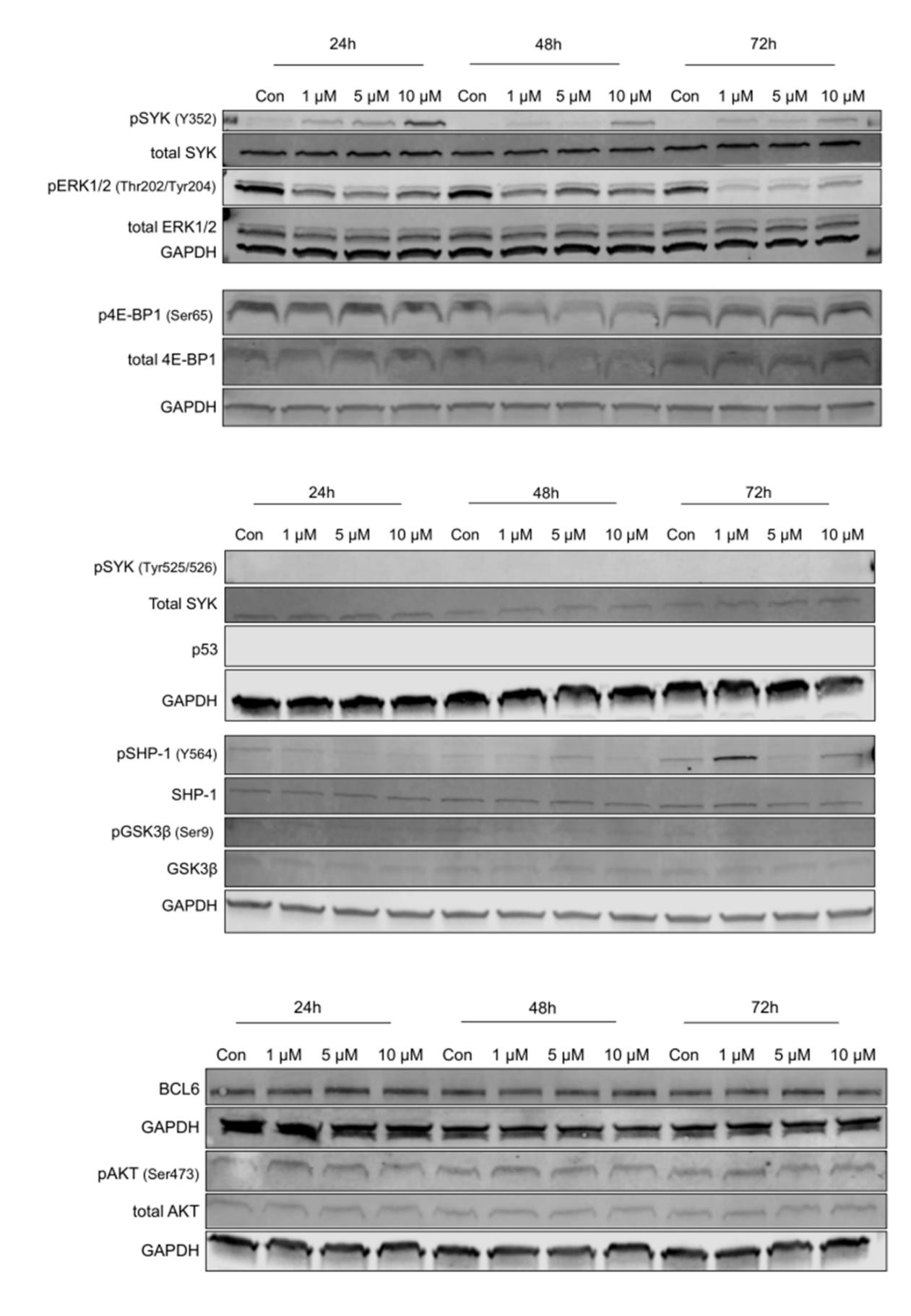


Figure S6: Entospletinib induced distinct downstream protein modifications in pre-B-ALL RS4;11

Western Blot analyses revealed distinct changes of SYK downstream proteins of in pro-B-ALL RS4;11. Representative Western Blot images of RS4;11 cells after 24 h, 48 h and 72 h Entospletinib exposure (n ≥ 3).
