# Supplementary material for: Precursor B-ALL Cell Lines Differentially Respond to SYK Inhibition by Entospletinib
Source: Int J Mol Sci. 2021 Jan 8;22(2):592. doi: 10.3390/ijms22020592 (PMC7827334; doi:10.3390/ijms22020592)
Supplement: Supplementary file 1 [file ijms-22-00592-s001.zip › supplementary material/Supplementary file C - methods.docx]

# Supplementary file C

# Materials and Methods

**Antibodies**

Table S1: Western Blot primary antibodies

| **Primary antibody** | **Order number** | **Manufacturer** |
| --- | --- | --- |
| pSYK (Tyr525/526) (C87C1) | rabbit #2710 | Cell Signaling (Danvers, MA, USA) |
| phospho-ZAP (Y319) / phospho-SYK (Y352) | rabbit #2701 | Cell Signaling (Danvers, MA, USA) |
| total SYK (D3Z1E) | rabbit #13198 | Cell Signaling (Danvers, MA, USA) |
| phospho-AKT (Ser473) | rabbit #9271 | Cell Signaling (Danvers, MA, USA) |
| total AKT | rabbit #9272 | Cell Signaling (Danvers, MA, USA) Cell Signaling (Danvers, MA, USA) |
| phospho-GSK3β (Ser9) (5B3) | rabbit #9323 | Cell Signaling (Danvers, MA, USA) |
| total GSK3β (27C10) | rabbit #9452 | Cell Signaling (Danvers, MA, USA) |
| phospho-p44/42 MAPK (ERK1/2) (Thr202/Tyr204) (D13.14.4E) XP | rabbit #4370 | Cell Signaling (Danvers, MA, USA) |
| total p44/42 MAPK (ERK1/2) (137F5) | rabbit #4695 | Cell Signaling (Danvers, MA, USA) |
| BCL-6 (D4I2V) XP | rabbit #14895 | Cell Signaling (Danvers, MA, USA) |
| phospho-SHP-1 (Tyr564) (D11G5) | rabbit #8849 | Cell Signaling (Danvers, MA, USA) |
| total SHP-1 (C14H6) | rabbit #3759 | Cell Signaling (Danvers, MA, USA) |
| p53 (1C12) | mouse #2524 | Cell Signaling (Danvers, MA, USA) |
| GAPDH (ZG003) | mouse #39-8600 | Invitrogen (Carlsbad, CA, USA) |

Table S2: Western Blot secondary antibodies

| **Secondary antibody** | **Order number** | **Manufacturer** |
| --- | --- | --- |
| IRDye 680RD Goat anti-Rabbit 0,5mg | 926-68071 | LI-COR Biosciences (Lincoln, Nebraska, USA) |
| IRDye 800CW Goat anti-Rabbit 0,5mg | 926-32211 | LI-COR Biosciences (Lincoln, Nebraska, USA) |
| IRDye 680RD Goat anti-Mouse 0,5mg | 926-68070 | LI-COR Biosciences (Lincoln, Nebraska, USA) |
| IRDye 800CW Goat anti-Mouse 0,5mg | 926-32210 | LI-COR Biosciences (Lincoln, Nebraska, USA) |

Table S3: Immunofluorescence antibodies

| **Primary antibody** | **Order number** | **Manufacturer** |
| --- | --- | --- |
| SYK | sc-1240 | Santa Cruz (Texas, USA) |
| Goat anti-mouse IgG (H+L) Alexa Fluor Plus | A32723 | Thermo Fisher Scientific (Massachusetts, USA) |

Table S4: Conjugated primary antibodies for intracellular flow cytometry

| **Secondary antibody** | **Order number** | **Manufacturer** |
| --- | --- | --- |
| Anti-Syk pY348-PE, human  Clone: REA681 | 130-110-400 | Miltenyi Biotec (Bergisch Gladbach, Germany) |
| Anti-Syk-FITC, human  Clone: REA111 | 130-099-278 | Miltenyi Biotec (Bergisch Gladbach, Germany) |
| REA Control (I) antibodies – FITC  Clone: REA293 | 130-104-611 | Miltenyi Biotec (Bergisch Gladbach, Germany) |
| REA Control (I) antibodies – PE  Clone; REA293 | 130-104-613 | Miltenyi Biotec (Bergisch Gladbach, Germany) |
